# Expression of immune checkpoints (IDO and PD-L1) in oral tongue cancer patients: a 10-year retrospective cohort study in Pakistan

**DOI:** 10.3389/fonc.2025.1495722

**Published:** 2025-08-12

**Authors:** Merium Fatima, Shaarif Bashir, Syed Atif Raza, Alamgeer, Muhammad Hassan, Muhammad Abu Bakar, Ali Zafar Sheikh, Muhammad Tahseen, Umer Nisar Sheikh, Asif Loya, Muhammad Faisal, Asim Farooq, Kashif Asghar

**Affiliations:** ^1^ Department of Pharmacy, Shaukat Khanum Memorial Cancer Hospital and Research Centre, Lahore, Pakistan; ^2^ Punjab University College of Pharmacy, University of The Punjab, Lahore, Pakistan; ^3^ Department of Pathology, Shaukat Khanum Memorial Cancer Hospital and Research Centre, Lahore, Pakistan; ^4^ Basic Sciences Research, Shaukat Khanum Memorial Cancer Hospital and Research Centre, Lahore, Pakistan; ^5^ Department of Cancer Registry and Clinical Data Management, Shaukat Khanum Memorial Cancer Hospital and Research Centre, Lahore, Pakistan; ^6^ Department of Radiology, Shaukat Khanum Memorial Cancer Hospital and Research Centre, Lahore, Pakistan; ^7^ Department of Surgical Oncology, Shaukat Khanum Memorial Cancer Hospital and Research Centre, Lahore, Pakistan

**Keywords:** tongue squamous cell carcinoma, IDO, PD-L1, chemotherapy, immunohistochemistry, biomarkers, immunotherapy

## Abstract

**Background:**

Tongue squamous cell carcinoma (TSCC) is a significant global health issue with high incidence and mortality rates. Current treatments involve surgery, radiotherapy, and chemotherapy; however, prognosis remains poor. Recent research highlights the crucial role of the tumor microenvironment, especially immune cells and checkpoints like PD-L1 and IDO, in TSCC progression.

**Aim:**

This study aims to investigate the expression of IDO and PD-L1 in TSCC patients before and after chemotherapy and their association with patients’ clinicopathological characteristics.

**Materials and Methods:**

This study involved 106 TSCC patients from Shaukat Khanum Memorial Cancer Hospital and Research Centre (SKMCH&RC) in Pakistan, with biopsies obtained from 2012 to 2022. Immunohistochemical analysis was performed on formalin-fixed, paraffin-embedded (FFPE) tumor samples to evaluate IDO and PD-L1 expression before and after chemotherapy. Data on patient demographics, tumor characteristics, and treatment were collected, and follow-up continued until January 2024.

**Results:**

The cohort had a mean age of 48.9 years, with a predominance of male patients. Prior to chemotherapy, 83% of patients were IDO-negative, and 75.5% were PD-L1-negative. Post-chemotherapy, IDO expression increased to 24.5% of patients (n = 26), with 84.6% exhibiting low expression and 15.4% showing high expression. While PD-L1 expression was increased to 29.2% (n = 31), with 90.3% of the positive cases showing low expression and 9.7% high expression. IDO expression was notably higher in multifocal tumors and correlated with increased comorbidities post-chemotherapy. Despite changes in marker expression, there was no significant difference in survival rates associated with IDO or PD-L1 expression.

**Conclusion:**

Chemotherapy appears to upregulate IDO and PD-L1 expressions in TSCC, highlighting the potential for integrating immunotherapy into treatment regimens. Further studies are needed to explore the dynamics of these biomarkers over time and their impact on patient outcomes, emphasizing the need for comprehensive therapeutic strategies.

## Introduction

Oral cancer is one of the most prevalent malignancies of the head and neck region, posing significant challenges to patient well-being and healthcare systems (1). The recent data from the Globocan 2022 report reveals the extensive impact of oral cancer, with a global incidence reaching 389,846 cases and a mortality toll of 188,438 (2). Specifically in Pakistan, oral cancer holds the position of the second most prevalent tumor, contributing to a substantial burden with 15,915 reported cases and 10,181 associated deaths (3). Among head and neck cancer cases, oral cancer holds the leading position with a continuing rise in its incidence (1). Oral cancer is characterized by its highly malignant nature, marked by elevated rates of local recurrence and cervical lymph node metastasis (4, 5). The reported 5-year survival rate for oral cancer is approximately 50% (6). Currently, the preferred treatment for tongue cancer involves a combination of surgery, postoperative radiotherapy, and chemotherapy (7). However, the prognosis for oral cancer remains grim due to short-term recurrence and inadequate therapeutic efficacy, significantly impacting the quality of life for affected patients (8).

Increasing evidence highlights the pivotal role played by the interaction between tongue cancer cells and the surrounding microenvironment in the development and prognosis of tongue cancer (9). Predominantly, TSCC stands out for its marked infiltration of immune cells, characterizing it as an immunogenic tumor (10). Among the prevalent stromal components in tongue cancer, the infiltrated immune cells emerge as key contributors to the carcinogenic process (10).

The concept of cancer immunoediting is widely accepted, and tumor immune escape is considered an emerging hallmark of cancer (10). Cancer immunotherapy utilizing immune-checkpoint inhibitors has emerged as a highly effective therapeutic modality for various cancers, including tongue cancer (11–13). In several malignancies, programmed death ligand-1 (PD-L1) checkpoint blockades have shown substantial clinical efficacy (14–16). Recent evidence has pointed out that the expression of PD-1 and PD-L1 were considerably associated with local recurrence in patients with TSCC (13). Overexpression of PD-L1 on tumor cells inhibits the activation of T cells, leading to the progression of tumors (17–20). PD-L1 overexpression in TSCC correlates with advanced stage and shorter disease-free survival (21). Several ongoing clinical trials have demonstrated efficacy against recurrent/metastatic head and neck squamous cell carcinoma (HNSCC), leading to clinical approval in multiple countries following successful phase III trials (22). Thus, the focus on immune checkpoints represents a new area of investigation for novel cancer therapies (23–25). Another such prospective target in this realm is indoleamine 2,3-dioxygenase (IDO), a checkpoint protein influencing an immunosuppressive tumor microenvironment (26). IDO, a heme-containing enzyme, metabolizes L-tryptophan into kynurenine (27). The localized depletion of tryptophan hinders T-cell cytotoxicity, thereby inhibiting T-cell immune responses through the induction of regulatory T-cell differentiation (28, 29). IDO expression has been reported in many cancers, including breast, colorectal, ovarian, gastric, and oral cancer (28, 30–34). It is also associated with cancer poor prognosis in oral squamous cell carcinoma patients (33). The significance of IDO expression is underscored by its ability to predict tumor responsiveness to anti-IDO immunotherapy. Similarly, high PD-L1 expression is associated with an unfavorable prognosis in diverse tumors. Thus, evaluating the levels of IDO and PD-L1 expressions may offer insights into identifying patients who could potentially benefit from anti-IDO/anti-PD-L1 therapy or combination therapies. In this study’s context, we performed an immunohistochemical analysis to investigate the expressions of IDO and PD-L1 in individuals with TSCC.

## Materials and methods

### Patients and data

We conducted a retrospective cross-sectional analysis involving tongue cancer patients who were registered at Shaukat Khanum Memorial Cancer Hospital and Research Centre (SKMCH&RC) in Pakistan. The study cohort comprised (106) patients diagnosed with tongue cancer between 2012 and 2022. Biopsy samples were collected from all patients before chemotherapy at the time of their initial diagnosis. Following neoadjuvant chemotherapy, all patients underwent surgical resection, during which post-treatment tissue samples were obtained for comparative expression analysis. Following surgery, all patients received radiotherapy, with some also receiving concurrent chemoradiotherapy (CRT); however, they developed metastases at a later stage. Since the primary objective was to evaluate the effect of chemotherapy, biomarker expression was analyzed only before and after chemotherapy. The impact of radiotherapy on expression levels was not assessed. These patients also underwent surgery following chemotherapy, but they developed metastases at a later stage.

Moreover, all patients received cisplatin plus gemcitabine (GC) as neoadjuvant chemotherapy. The use of GC was based on prior experience at SKMCH&RC, Lahore, Pakistan. A previous study from our institution demonstrated that GC was well tolerated, had low toxicity, and showed significant antitumor activity in locally advanced head and neck cancer (HANC) (35). Building on these positive results and ongoing clinical success, GC has been adopted as the institutional neoadjuvant regimen for selected cases of advanced HANC.

Biopsies stored in formalin-fixed, paraffin-embedded (FFPE) blocks were obtained from the pathology department at SKMCH&RC. Extensive patient data, including demographics, pathological and radiological characteristics, as well as treatment specifics, were retrieved from SKMCH&RC’s electronic medical records system. Patient follow-up for survival analysis was extended until Jan 2024. The study received approval from the institutional review board (IRB) of SKMCH&RC (EX-05-12-22-02), with the IRB granting a waiver of informed consent due to the minimal risk posed to patients’ rights, safety, and well-being, given that the data and FFPE samples originated from archived records.

### PD-L1 and IDO Expression analysis by immunohistochemistry

Two sections of FFPE tumor specimens of the same patients were cut at a thickness of 4 µm. IDO staining was performed using an anti-Indoleamine 2, 3-dioxygenase antibody (Cat # 86630S IDO (D5J4E™) Rabbit mAb); heat-mediated epitope retrieval with a Tris-EDTA buffer was performed. The immunoreactivity was detected by using the Dako EnVision kit (K8002). Normal human reactive lymph nodes served as a positive control. PD-L1 immunoreactivity was assessed by an immunohistochemical assay for FFPE tumor specimens. Slides were stained using an autostainer Link 48 (Dako Denmark) as per the manufacturer’s protocol. Slides were deparaffinized and antigen was retrieved simultaneously with the target retrieval low pH solution (#GV8005 Dako). PD-L1 antibody (22C3) and an automated staining procedure developed by DAKO. PD-L1 labeling was visualized using the Envision Flex detection kit DAKO (K8002). Normal human tonsils served as a positive control. Slides were visualized by an optical microscope (Provis AX-70, Olympus, Melville, NY).

### Scoring

Pathologists assessed all the results. They performed a blind histopathologic evaluation. The discrepancies between the pathologists were examined mutually to reach a consensus and the mean score of both was considered a decisive score. The total IDO immunostaining scores were calculated as described earlier (36). The intensity was scored for IDO as negative (0), weak (1), moderate (2), or strong (3). The percentage of positive tumor cells was classified into four categories: diffuse (3+, 50–75%), focal (2+, 25–50%), sporadic (1+, 5–25%), and negative (0, 0%). The immunohistochemical expression of PD-L1 was calculated as described earlier (37). PD-L1 staining intensity was assessed as strong (3), moderate (2), weak (1), or negative (0). The percentage of tumor cells with positive staining was categorized according to the following formula: PD-L1 expression score (H score) (range, 0–9)=0×% of non-stained tumor cells +1×% of weakly stained tumor cells +2×% of moderately stained tumor cells +3×% of strongly stained tumor cells.

Currently CPS scoring is the standard practice in PD-L1 interpretation of Oral SCCs (38–40). The CPS scoring system was used to ensure uniformity and comparability with scoring system used for IDO IHC. The CPS score was used as a cut off for positive results and then as an additional step, the intensity of stain and overall percentage of positive cells were also considered (38, 41–44). This approach was followed to harmonize our IDO results, as both intensity and percentage evaluation for IDO and PD-L1 were applied.

### Statistical analysis

Statistical analysis was performed by using SPSS software (version 20.0; SPSS, Chicago, IL, USA). Frequency and percentage were used for categorical variables while the median and range (min-max) were used for continuous variables. Bivariate analysis was done using chi-square or Fisher exact test (where necessary). For continuous explanatory variables such as age, the independent t-test was performed. In addition, McNemar’s test was performed to bifurcate the categorical pre and post data. Survival curves were generated using the Kaplan–Meier tool to estimate the probability of survival over time. Statistical significance was defined as a two-tailed P-value of 0.05.

## Results

### Patient demographics

The study included 106 participants with a mean age of 48.9 years (SD = 11.9) and a median age of 50.5 years, ranging from 20 to 78 years. Among the participants, 67.0% were male (n = 71) and 33.0% were female (n = 35). The demographic data reveals a predominantly middle-aged male cohort with a mean BMI (26.1 ± 4.5) in the overweight range. Most participants had no family history of disease or history of substance use. Most participants did not have comorbidities, but among those who did, hypertension was the most common condition (52.4%). Among 106 patients, 9.4% of tumors were poorly differentiated, 59.4% were moderately differentiated, and 31.1% were well differentiated as shown in **Table 1**. The majority of tumors in this cohort were moderately differentiated and primarily unifocal with uninvolved margins. The tumors exhibited a wide range in size and depth of invasion, with a notable proportion at advanced T stages. Despite extensive lymph node extraction, positive nodes were relatively few on average, although extra nodal extension, perineural invasion were significant concerns.

**Table 1:** Descriptive statistics of demographic and clinicopathological characteristics.

Variables	Characteristics	Total N = 106 (100.0%)
Age		
	Mean ± SD	48.9 ± 11.9
	Median (min-max)	50.5 (20–78)
Sex		
	Male	71 (67.0)
	Female	35 (33.0)
Body mass index (kg/m2)		
	Mean ± SD	26.1 ± 4.5
	Median (min-max)	25.3 (16.5-36.9)
Family History		
	Absent	92 (86.8)
	Present	14 (13.2)
History of Pan/Niswar		
	Absent	74 (69.8)
	Present	32 (30.2)
History of tobacco use		
	Absent	71 (67.0)
	Present	32 (30.2)
Comorbidities		
	Absent	85 (80.2)
	Present	21 (19.8)
	• DM	4 (19.0)
	• HTN	11 (52.4)
	• DM + HTN	5 (23.8)
	• DM + Hepatitis C	1 (4.8)
Differentiation		
	Poorly differentiated	10 (9.4)
	Moderately differentiated	63 (59.4)
	Well differentiated	33 (31.1)
Tumor site		
	Left	56 (52.8)
	Right	50 (47.2)
Focality		
	Single	104 (98.1)
	Bifocal	1 (0.9)
	Multifocal	1 (0.9)
Margin status		
	Uninvolved	103 (97.2)
	Involved	3 (2.8)
Tumor size (mm)		
	Mean ± SD	21.8 ± 11.3
	Median (min-max)	20.0 (2–65)
DOI (mm)		
	Mean ± SD	8.8 ± 4.7
	Median (min-max)	9.0 (1–21)
T & N Staging (Pre-CT)		
T Stage		
	0	0 (0)
	1	0 (0)
	2	10 (9.43)
	3	72 (67.92)
	4	24 (22.64)
N Stage		
	0	64 (60.37)
	1	29 (27.35)
	2	12 (11.32)
	3	1 (0.94)
	4	0 (0)
T & N Staging (Post-CT)		
T stage		
	0	1 (0.9)
	1	25 (23.6)
	2	45 (42.5)
	3	32 (30.2)
	4	3 (2.8)
N stage		
	0	48 (45.3)
	1	17 (16.0)
	2	29 (27.4)
	3	12 (11.3)
	4	0 (0)
Total lymph node extracted		
	Mean ± SD	62.5 ± 30.4
	Median (min-max)	59.5 (0–174)
No. of positive lymph node		
	Mean ± SD	1.9 ± 3.2
	Median (min-max)	1.0 (0–16)
Extra nodal extension		
	Absent	90 (84.9)
	Present	16 (15.1)
Perineural invasion		
	Absent	87 (82.1)
	Present	19 (17.9)
Lymph vascular invasion		
	Absent	101 (95.3)
	Present	5 (4.7)
Recurrence		
	No	66 (62.3)
	Yes	40 (37.7)
Metastasis		
	No	93 (87.7)
	Yes	13 (12.3)
Status		
	Alive	23 (21.7)
	Death	63 (59.4)
	Unknown	20 (18.9)

DM, Diabetes mellitus; HTN, hypertension; CT, Chemotherapy; DOI, Depth of invasion.

### Immunohistochemical staining

**Figure 1:**
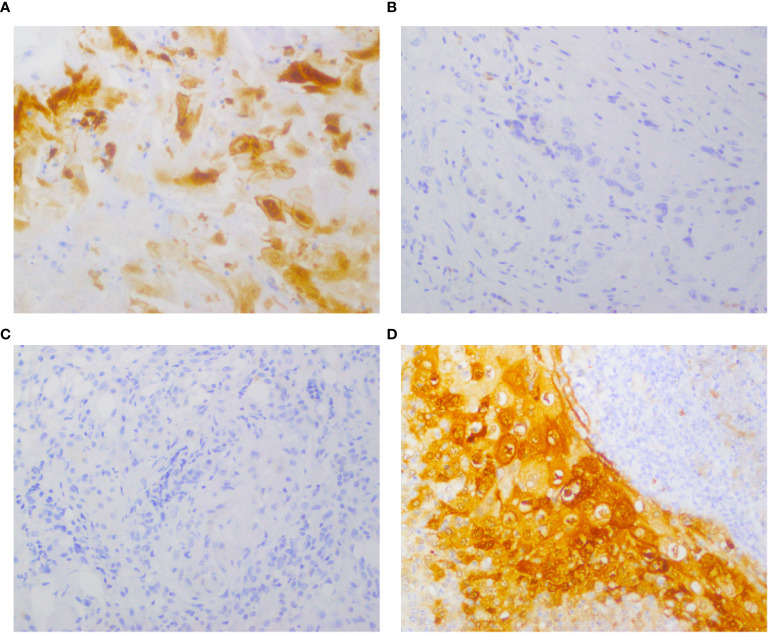
Expression of IDO before and after chemotherapy in the same patient, detected by immunohistochemical staining. Representative images of immunohistochemical staining for IDO in TSCC cases. **(A, B)** Positive IDO expression in tumor cells pre-chemotherapy; negative IDO expression post-chemotherapy. **(C, D)** Negative IDO expression pre-chemotherapy; positive IDO expression post-chemotherapy.

**Figure 2:**
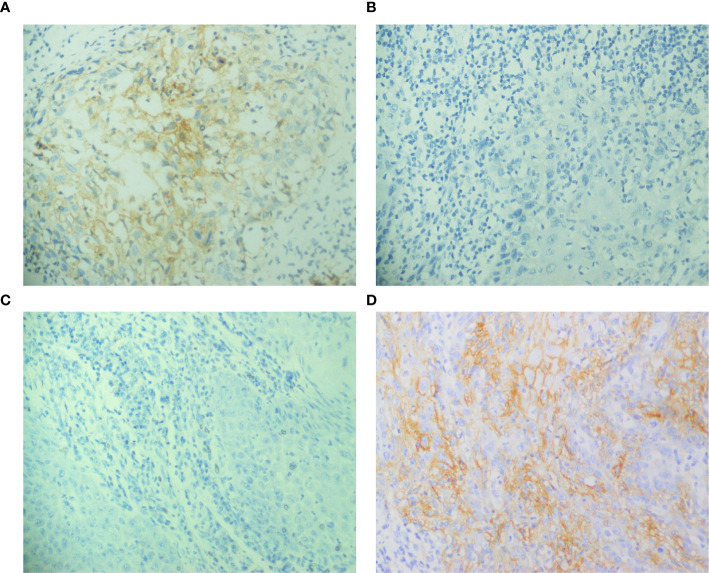
Expression of PD-L1 before and after chemotherapy in the same patient, detected by immunohistochemical staining. **(A, B)** Positive PD-L1 expression pre-chemotherapy; negative PD-L1 expression post-chemotherapy, **(C, D)** Negative PD-L1 expression pre-chemotherapy; positive PD-L1 expression post-chemotherapy.

**Figure 3:**
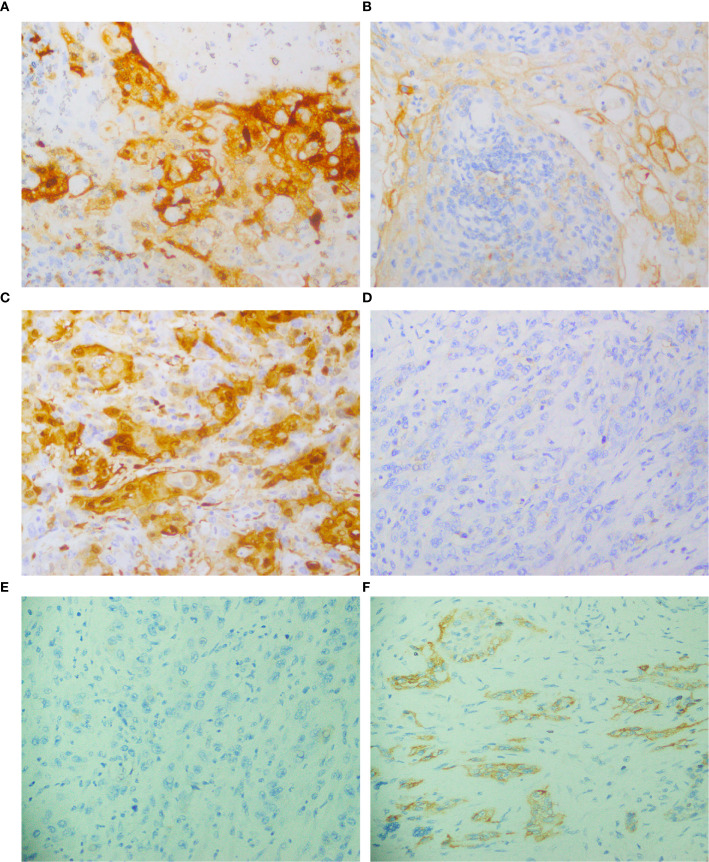
Expression of IDO and PD-L1 in the same patient, detected by immunohistochemical staining. **(A, B)** IDO positive expression; PD-L1 positive expression. **(C, D)** IDO positive expression; PD-L1 negative expression, **(E, F)** IDO negative expression; PD-L1 positive expression. All images were captured at 40X magnification.

The study assessed the expression of IDO and PD-L1 in 106 patients before and after chemotherapy. Prior to chemotherapy, IDO was negative in 83% of patients (n = 88) (**Figure 1C**) and positive in 17% (n = 18) (**Figure 1A**). Of the positive cases, 81.3% had low expression, while 16.7% had high expression. Post-chemotherapy, IDO expression increased to 24.5% of patients (n = 26), with 84.6% exhibiting low expression and 15.4% showing high expression (**Figure 1D**). Among those who were initially IDO positive, 38.9% (n = 7) became IDO negative (**Figure 1B**), and 61.1% (n = 11) remained positive. For PD-L1, prior to chemotherapy, 75.5% of patients (n = 80) were negative (**Figure 2C**) and 24.5% (n = 26) were positive (**Figure 2A**). Among the positive cases, 88.5% had low expression and 11.5% had high expression. After chemotherapy, the PD-L1 expression increased to 29.2% (n = 31), with 90.3% of the positive cases showing low expression and 9.7% high expression (**Figure 2D**). Our dataset shows that IDO and PD-L1 expressions increased after chemotherapy, suggesting that chemotherapy may upregulate these markers. The negative expression of both markers remain predominant (**Figures 3D, E**) though there is a slight increase in high expression cases post-chemotherapy (**Figures 3A–C, F**).

### Clinicopathological associations with IDO expression prior to chemotherapy

The study analyzed 106 patients to investigate the relationship between demographic and clinicopathological characteristics and IDO status before chemotherapy. A significant association was found between IDO positivity and PD-L1 positivity (p = 0.001), with 50% of IDO-positive patients also being PD-L1 positive. This indicates an immunosuppressive microenvironment in this subset of patients. No significant associations were observed between IDO status and factors such as age, sex, or tumor size. Additionally, we found that all multifocal tumors had positive IDO expression (p = 0.02), as shown in **Table 2**.

**Table 2:** Demographic and clinicopathological characteristics versus IDO (before chemotherapy) negative and positive.

Variables	Characteristics	Negative 88 (83.0%)	Positive 18 (17.0%)	p-value
PD-L1 before chemotherapy				0.001
	Negative	71 (80.7)	9 (50.0)	
	Positive	17 (19.3)	9 (50.0)	
Age				0.38
	Mean ± SD	48.4 ± 12.1	51.2 ± 10.9	
Sex				0.58
	Male	59 (67.0)	12 (66.7)	
	Female	29 (33.0)	6 (33.3)	
Body mass index (kg/m^2^)				0.70
	Mean ± SD	26.0 ± 4.4	26.5 ± 5.2	
Family History				0.70
	Absent	77 (87.5)	15 (83.3)	
	Present	11 (12.5)	3 (16.7)	
History of Pan/Niswar				0.57
	Absent	60 (68.2)	14 (77.8)	
	Present	28 (31.8)	4 (22.2)	
History of tobacco use				0.59
	Absent	60 (68.2)	11 (61.1)	
	Present	28 (31.8)	7 (38.9)	
Comorbidities				0.52
	Absent	69 (78.4)	16 (88.9)	
	Present	19 (21.6)	2 (11.1)	
	• DM	4 (21.1)	–	
	• HTN	10 (52.6)	1 (50.0)	
	• DM + HTN	4 (21.1)	1 (50.0)	
	• DM + Hepatitis C	1 (5.3)	–	
Differentiation				0.28
	Poorly differentiated	8 (9.1)	2 (11.1)	
	Moderately differentiated	55 (62.5)	8 (44.4)	
	Well differentiated	25 (28.4)	8 (44.4)	
Tumour site				0.19
	Left	44 (50.0)	12 (66.7)	
	Right	44 (50.0)	6 (33.3)	
Focality				0.02
	Single	88 (100.0)	16 (88.9)	
	Bifocal	–	1 (5.6)	
	Multifocal	–	1 (5.6)	
Margin status				1.00
	Uninvolved	85 (96.6)	18 (100.0)	
	Involved	3 (3.4)	–	
Tumour size (mm)				0.96
	Mean ± SD	21.5 ± 11.7	21.7 ± 10.2	
DOI (mm)				0.74
	Mean ± SD	8.7 ± 4.8	9.2 ± 3.9	
T stage				0.37
	0	1 (1.1)	–	
	1	21 (23.9)	4 (22.2)	
	2	40 (45.5)	5 (27.8)	
	3	24 (27.3)	8 (44.4)	
	4	2 (2.3)	1 (5.6)	
N stage				
	0	42 (47.7)	6 (33.3)	
	1	13 (14.8)	4 (22.2)	
	2	22 (25.0)	7 (38.9)	
	3	11 (12.5)	1 (5.6)	
Recurrence				0.67
	No	54 (61.4)	12 (66.7)	
	Yes	34 (38.6)	6 (33.3)	
Metastasis				0.11
	No	75 (85.2)	18 (100)	
	Yes	13 (14.8)	0 (0)	
Total lymph node extracted				0.34
	Mean ± SD	61.2 ± 27.0	68.7 ± 43.3	
No. of positive lymph node				0.67
	Mean ± SD	2.0 ± 3.4	1.7 ± 2.2	
Extra nodal extension				0.46
	Absent	76 (86.4)	14 (77.8)	
	Present	12 (13.6)	4 (22.2)	

DM, Diabetes mellitus; HTN, hypertension; DOI, depth of invasion.

### Clinicopathological associations with IDO expression post chemotherapy

Post-chemotherapy, we observed a continued significant association between IDO positivity and PD-L1 status (p = 0.001). Additionally, IDO-positive patients had more comorbidities (p = 0.03). No significant differences were found for age, sex, BMI, family history, tobacco use, histology, tumor site, margin status, tumor size, depth of invasion, T stage, N stage, positive lymph nodes, and extranodal extension, as shown in **Table 3**.

**Table 3:** Demographic and clinicopathological characteristics versus IDO (after chemotherapy) negative and positive.

Variables	Characteristics	Negative 80 (75.5%)	Positive 26 (24.5%)	p-value
PD-L1 after chemotherapy				0.001
	Negative	62 (77.5)	13 (50.0)	
	Positive	18 (22.5)	13 (50.0)	
Age				0.13
	Mean ± SD	47.9 ± 11.7	51.9 ± 12.5	
Sex				0.34
	Male	56 (70.0)	15 (57.7)	
	Female	24 (30.0)	11 (42.3)	
Body mass index (kg/m^2^)				0.57
	Mean ± SD	25.9 ± 4.4	26.5 ± 4.9	
				
Family History				1.00
	Absent	69 (86.3)	23 (88.5)	
	Present	11 (13.8)	3 (11.5)	
History of Pan/Niswar				0.81
	Absent	55 (68.8)	19 (73.1)	
	Present	25 (31.3)	7 (26.9)	
History of tobacco use				0.34
	Absent	56 (70.0)	15 (57.7)	
	Present	24 (30.0)	11 (42.3)	
Comorbidities				0.03
	Absent	67 (83.8)	18 (69.2)	
	Present	13 (16.3)	8 (30.8)	
	• DM	4 (30.8)	–	
	• HTN	5 (38.5)	6 (75.0)	
	• DM + HTN	4 (30.8)	1 (12.5)	
	• DM + Hepatitis C	–	1 (12.5)	
Differentiation				0.52
	Poorly differentiated	7 (8.8)	3 (11.5)	
	Moderately differentiated	50 (62.5)	13 (50.0)	
	Well differentiated	23 (28.7)	10 (38.5)	
Tumour site				0.82
	Left	43 (53.8)	13 (50.0)	
	Right	37 (46.3)	13 (50.0)	
Focality				0.05
	Single	80 (100.0)	24 (92.3)	
	Bifocal	–	1 (3.8)	
	Multifocal	–	1 (3.8)	
Margin status				1.00
	Uninvolved	77 (96.3)	26 (100.0)	
	Involved	3 (3.8)	–	
Tumour size (mm)				0.63
	Mean ± SD	21.8 ± 11.9	20.6 ± 9.9	
DOI (mm)				0.76
	Mean ± SD	8.7 ± 4.6	9.1 ± 4.8	
T stage				0.76
	0	1 (1.3)	–	
	1	19 (23.8)	6 (23.1)	
	2	32 (40.0)	13 (50.0)	
	3	26 (32.5)	6 (23.1)	
	4	2 (2.5)	1 (3.8)	
N stage				0.17
	0	38 (47.5)	10 (38.5)	
	1	9 (11.3)	8 (30.8)	
	2	23 (28.7)	6 (23.1)	
	3	10 (12.5)	2 (7.7)	
Recurrence				0.39
	No	48 (60.0)	18 (69.2)	
	Yes	32 (40.0)	8 (30.0)	
Metastasis				0.51
	No	69 (86.3)	24 (92.3)	
	Yes	11 (13.8)	2 (7.7)	
Total lymph node extracted				0.83
	Mean ± SD	62.8 ± 28.1	61.4 ± 36.8	
No. of positive lymph node				0.14
	Mean ± SD	2.2 ± 3.6	1.2 ± 1.3	
Extra nodal extension				0.76
	Absent	67 (83.8)	23 (88.5)	
	Present	13 (16.3)	3 (11.5)	

DM, Diabetes mellitus; HTN, hypertension; DOI, depth of invasion.

### Impact of chemotherapy on IDO and PD-L1 expression

In our dataset, 18 patients were IDO positive prior to chemotherapy. After chemotherapy, 7 of these patients became IDO negative. Conversely, among the 88 patients who were initially IDO negative, 15 became IDO positive, resulting in a total of 26 IDO-positive patients’ post-chemotherapy. This change in IDO expression was statistically significant (p = 0.04) according to McNemar’s test (**Table 4**). Chemotherapy significantly impacted IDO expression, with a notable proportion of initially IDO-positive patients maintaining their positive status post-treatment and some initially IDO-negative patients becoming positive.

**Table 4:** Impact of chemotherapy on IDO expression before and after.

Variables	Characteristics	IDO before chemotherapy		p-value
		Negative 88 (83.0%)	Positive 18 (17.0%)	
IDO after chemotherapy				0.04*
	Negative	73 (83.0)	7 (38.9)	
	Positive	15 (17.0)	11 (61.1)	

*McNemar’s test.

Similarly, in our dataset, 26 patients were PD-L1 positive prior to chemotherapy (**Table S1** in **Supplementary Material**). After treatment, 14 of these patients became PD-L1 negative (**Figure 2B**). Conversely, among the 80 patients who were initially PD-L1 negative, 19 became positive, resulting in a total of 31 PD-L1-positive patients’ post-chemotherapy (**Table S2** in **Supplementary Material**). The change in PD-L1 expression approached statistical significance (p = 0.09) according to McNemar’s test (**Table 5**). Chemotherapy demonstrated a trend towards altering PD-L1 expression, with some patients switching their PD-L1 status post-treatment. Although the change did not reach statistical significance, the trend may suggest that chemotherapy may influence PD-L1 expression, potentially impacting the tumor’s immune evasion capabilities.

**Table 5:** Impact of chemotherapy on PD-L1 expression before and after.

**Variables**	**Characteristics**	**PD-L1 before chemotherapy**		**p-value**
		**Negative** **80 (75.5%)**	**Positive** **26 (24.5%)**	
PD-L1 after chemotherapy				0.09*
	Negative	61 (76.3)	14 (53.8)	
	Positive	19 (23.8)	12 (46.2)	

*McNemar’s test.

### Survival analysis

**Figure 4:**
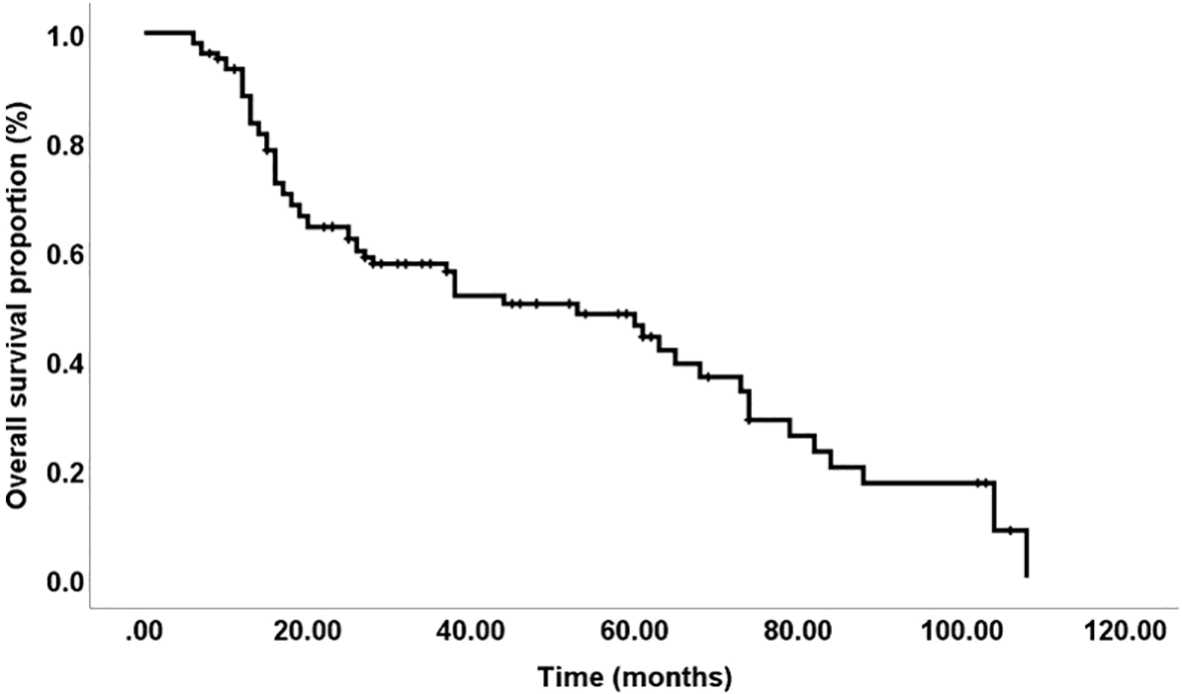
Overall survival: The five-year disease-free survival rate was 49%, with a median duration of 53 months.

In our study, 23 patients (21.7%) were alive at the time of last follow-up, while 63 patients (59.4%) had died while 20 (18.9) were unknown. The overall five-year survival rate was 49%, with a median survival time of 53 months, as depicted in **Figure 4**. We found no significant differences in survival associated with IDO and PD-L1 expression. Pre-chemotherapy, IDO-positive patients had a 68% five-year survival rate compared to 42% for IDO-negative patients (p = 0.18). Post-chemotherapy, these rates were 55% for IDO-positive and 43% for IDO-negative (p = 0.43). For PD-L1 expression, pre-chemotherapy survival was 51% for positive versus 44% for negative (p = 0.86), and post-chemotherapy, it was 45% for positive versus 47% for negative (p = 0.64).

## Discussion

TSCC is one of the most prevalent malignancies of the head and neck region, constituting a significant global health concern with a survival rate of about 50% (45–47). Traditionally managed with surgery and radiotherapy or chemoradiotherapy, recent research has focused on immunotherapy to improve outcomes (47). The response rate to PD-1 and PD-L1 antibody treatment remains limited, with only about 20% of patients showing favorable outcomes in most solid tumors (48). PD-L1 is a transmembrane glycoprotein structurally characterized by extracellular IgV- and IgC-like domains (49). Under physiological conditions, PD-L1 is expressed by various immune cells, including macrophages, activated T and B lymphocytes, dendritic cells, and certain epithelial cells, particularly in response to pro-inflammatory signals (50). Within the tumor microenvironment, PD-L1 is frequently overexpressed on malignant cells as an adaptive mechanism to suppress cytotoxic T-cell activity and facilitate immune escape (51). This upregulation is often observed in immune-infiltrated tumors characterized by a high density of CD8+ T cells, Th1 cytokines, interferons, and distinct immunomodulatory gene signatures (52). Multiple oncogenic and inflammatory pathways regulate PD-L1 expression in digestive system cancers including the EGFR/ERK signaling axis in esophageal squamous cell carcinoma (53), the JAK/STAT pathway in gastric cancer (54), and the ERK/MAPK pathway in hepatocellular carcinoma (55). In addition, the IFN-γ–driven activation of the JAK/STAT pathway has been shown to mediate PD-L1 induction in myeloid leukemia as well as pancreatic and gastric cancers (56, 57).

Similarly, another potential focus is on IDO, a checkpoint protein implicated in creating an immunosuppressive environment within tumors (58). IDO is a cytosolic heme-containing enzyme that catalyzes the first and rate-limiting step in the metabolism of tryptophan (Trp) to kynurenine (Kyn) (59). It initiates the oxidative cleavage of the pyrrole ring of L-tryptophan to produce N-formylkynurenine, which is subsequently converted into L-kynurenine by kynurenine formamidase (60). Kynurenine and its downstream metabolites contribute to immunosuppression by inhibiting T-cell responses, enabling tumor cells to evade immune surveillance (61). L-kynurenine is further metabolized to anthranilic acid via kynureninase, then to 3-hydroxyanthranilic acid through kynurenine-3-monooxygenase, ultimately generating 2-amino-muconic acid, picolinic acid, and quinolinic acid (62). These downstream metabolites reinforce immune evasion by promoting regulatory T-cell differentiation and inhibiting effector T-cell activity, thereby fostering an immunosuppressive tumor microenvironment (63).

IDO plays a critical role in the pathogenesis of various conditions, including chronic inflammatory diseases, infections, and a wide range of malignancies (33, 64, 65). Its aberrant overexpression has been reported in several tumor types, including oral, colorectal, hepatocellular, ovarian cancers, and melanomas (33, 65). High IDO expression within the tumor microenvironment contributes to immune evasion, facilitates tumor progression and metastasis, and is strongly associated with poor clinical outcomes and decreased overall survival in cancer patients (66–68). Consistently, preclinical studies have demonstrated that IDO overexpression is linked to poor prognosis across multiple cancer types (69). Elevated IDO levels have been observed across various cancers such as breast, colorectal, ovarian, gastric cancer, and TSCC (30–34, 65), correlating with poorer prognosis in TSCC patients (70). This highlights the necessity for enhanced combined immunotherapeutic strategies and predictive biomarkers to effectively identify patients who could derive optimal benefit from these treatments.

In this study, we evaluated the expression of IDO and PD-L1 in 106 patients with tongue cancer both before and after chemotherapy. Our analysis indicates that chemotherapy significantly influences the expression of IDO and PD-L1. Prior to chemotherapy, the majority of patients were negative for IDO (83%) and PD-L1 (75.5%). Post-chemotherapy, there was an increase in the expression of both markers, with IDO positivity rising to 24.5% and PD-L1 positivity to 29.2%. This suggests that chemotherapy may upregulate these immunosuppressive markers, potentially impacting the tumor microenvironment and immune evasion mechanisms. The increase in IDO and PD-L1 expression post-chemotherapy could imply a chemoresistance mechanism, where tumors adapt to therapeutic pressures by enhancing immunosuppressive pathways.

Regarding the effect of chemotherapy on PD-L1 expression, several studies have documented changes in PD-L1 expression following traditional cancer treatments like chemotherapy, radiotherapy, or their combination. In a study conducted by Park BJ et al, chemoradiation therapy has shown to significantly alter PD-L1 expression in locoregional recurrent squamous cell carcinomas of the head and neck (71). This change in PD-L1 score post-treatment suggests a potential shift in the tumor’s immune microenvironment, which may influence the effectiveness of subsequent immunotherapeutic approaches (71). The findings highlight the importance of re-assessing PD-L1 status in recurrent tumors, as initial evaluations conducted prior to treatment may not accurately represent the post-therapy immune profile. A systematic review by Van den Ende et al. identified 48 studies examining PD-L1 expression changes post-treatment. Statistical analysis indicated that 30 of these studies reported increased PD-L1 expression in post-treatment specimens or treated versus untreated samples (72). However, Karpathiou et al. evaluated the stability of PD-L1 expression in 106 HNSCC tissue specimens over storage periods of 20–48 months (73). They reported a significant decline in PD-L1 tumor proportional score, immune cell expression, and combined positive score over time, indicating loss of antigenicity in archived FFPE samples (73). This degradation may lead to underestimation of PD-L1 expression, potentially influencing treatment decisions and study results depending on the interval between the first and second biopsy. Timely evaluation on freshly processed tissue is therefore recommended for accurate immunotherapy eligibility assessment.

Despite an extensive literature search, we found no studies addressing IDO status before and after chemotherapy specifically in oral squamous cell carcinoma. However, our findings align with other research indicating elevated IDO expression post-chemotherapy (74). IDO-inducing signals may be inherently present in the tumor’s inflammatory microenvironment and could be further triggered by dying cells and tumor antigens released during chemotherapy. Nevertheless, the precise extent of IDO production following chemotherapy remains largely unexplored (74).

Our study found a significant association between IDO and PD-L1 positivity both before and after chemotherapy, reinforcing the notion of an immunosuppressive microenvironment in IDO-positive patients. Multifocal tumors were also significantly associated with IDO positivity prior to chemotherapy, suggesting a potential link between tumor multiplicity and immunosuppressive marker expression. Post-chemotherapy, IDO-positive patients had more comorbidities, highlighting the complex interplay between patient health status and tumor biology.

The overall five-year survival rate for patients in this study was 49%, with a median survival time of 53 months. Despite investigating IDO and PD-L1 expressions, our analysis did not reveal significant differences in survival outcomes related to these markers. This finding suggests that, while IDO and PD-L1 are involved in the immunosuppressive environment of TSCC, their expression levels may not directly impact overall survival within this cohort. Further research could explore additional factors influencing survival and assess whether combining IDO and PD-L1 with other biomarkers might provide a more comprehensive understanding of patient prognosis.

To the best of our knowledge, no study has yet examined the expression of both biomarkers before and after chemotherapy. This highlights the strength of our research, providing valuable insights into the immunosuppressive dynamics within oral squamous cell carcinoma before and after chemotherapy. Future research should focus on longitudinal studies to monitor these markers over time. Additionally, further investigation into the molecular pathways driving these expression changes could uncover novel therapeutic targets.

The current study has some limitations. The retrospective design and reliance on existing patient records may introduce selection bias and limit data quality control. The cross-sectional analysis only captures data at two points, missing dynamic changes. A larger cohort would enhance statistical power and overall impact of the study.

## Conclusion

The current study demonstrates that chemotherapy increases IDO and PD-L1 expression in TSCC, suggesting that incorporating immunotherapy could benefit patients undergoing chemotherapy. Future research should emphasize longitudinal studies and the combinatorial analysis of multiple biomarkers to gain a clearer understanding of immune marker expression dynamics in TSCC.

## Data Availability

The original contributions presented in the study are included in the article/**Supplementary Material**. Further inquiries can be directed to the corresponding authors.
