# Supplementary material for: Expression of immune checkpoints (IDO and PD-L1) in oral tongue cancer patients: a 10-year retrospective cohort study in Pakistan
Source: Front Oncol. 2025 Aug 12;15:1495722. doi: 10.3389/fonc.2025.1495722 (PMC12378751; doi:10.3389/fonc.2025.1495722)
Supplement: Supplementary file 1 [file Table1.docx]

**Table S1: Demographic and clinicopathological characteristics versus PD-L1 (before chemotherapy) negative and positive.**

| Variables | Characteristics | Negative  80 (75.5%) | Positive  26 (24.5%) | p-value |
| --- | --- | --- | --- | --- |
| IDO before chemotherapy | |  |  | 0.01 |
|  | Negative | 71 (88.8) | 17 (65.4) |  |
|  | Positive | 9 (11.3) | 9 (34.6) |  |
| Age |  |  |  | 0.38 |
|  | Mean ± SD | 48.7 ± 12.4 | 49.3 ± 10.5 |  |
| Sex |  |  |  | 0.84 |
|  | Male | 54 (67.5) | 9 (34.6) |  |
|  | Female | 26 (32.5) | 17 (65.4) |  |
| Body mass index (kg/m^2^) |  |  |  | 0.92 |
|  | Mean ± SD | 26.0 ± 4.4 | 26.1 ± 4.7 |  |
|  |  |  |  |  |
| Family History |  |  |  | 1.00 |
|  | Absent | 69 (86.3) | 23 (88.5) |  |
|  | Present | 11 (13.8) | 3 (11.5) |  |
| History of Pan/Niswar |  |  |  | 0.94 |
|  | Absent | 56 (70.0) | 18 (69.2) |  |
|  | Present | 24 (30.0) | 8 (30.2) |  |
| History of tobacco use |  |  |  | 0.78 |
|  | Absent | 53 (63.3) | 18 (69.2) |  |
|  | Present | 27 (33.8) | 8 (30.8) |  |
| Comorbidities |  |  |  | 0.78 |
|  | Absent | 63 (78.8) | 22 (84.6) |  |
|  | Present | 17 (21.3) | 4 (15.4) |  |
|  | - DM | 4 (5.0) | - |  |
|  | - HTN | 8 (10.0) | 3 (11.5) |  |
|  | - DM + HTN | 4 (5.0) | 1 (3.8) |  |
|  | - DM + Hepatitis C | 1 (1.3) | - |  |
| Differentiation |  |  |  | 0.21 |
|  | Poorly differentiated | 6 (7.5) | 4 (15.4) |  |
|  | Moderately differentiated | 46 (57.5) | 17 (65.4) |  |
|  | Well differentiated | 28 (35.0) | 5 (19.2) |  |
| Tumour site |  |  |  | 0.43 |
|  | Left | 44 (55.0) | 12 (46.2) |  |
|  | Right | 36 (45.0) | 14 (53.8) |  |
| Focality |  |  |  | 0.18 |
|  | Single | 79 (98.8) | 25 (96.2) |  |
|  | Bifocal | - | 1 (3.8) |  |
|  | Multifocal | 1 (1.3) | - |  |
| Margin status |  |  |  | 1.00 |
|  | Uninvolved | 77 (96.3) | 26 (100.0) |  |
|  | Involved | 3 (3.8) | - |  |
| Tumour size (mm) |  |  |  | 0.96 |
|  | Mean ± SD | 21.5 ± 11.7 | 21.6 ± 10.2 |  |
| DOI (mm) |  |  |  | 0.74 |
|  | Mean ± SD | 8.7 ± 4.8 | 9.1 ± 3.9 |  |
| T stage |  |  |  | 0.13 |
|  | 0 | - | 1 (3.8) |  |
|  | 1 | 22 (27.5) | 3 (11.5) |  |
|  | 2 | 35 (43.8) | 10 (38.5) |  |
|  | 3 | 21 (26.3) | 11 (42.3) |  |
|  | 4 | 2 (2.5) | 1 (3.8) |  |
| N stage |  |  |  | 0.06 |
|  | 0 | 42 (52.5) | 6 (23.1) |  |
|  | 1 | 12 (15.0) | 5 (19.2) |  |
|  | 2 | 19 (23.8) | 10 (38.5) |  |
|  | 3 | 7 (8.8) | 5 (19.2) |  |
| Recurrence |  |  |  | 0.81 |
|  | No | 49 (61.3) | 17 (65.4) |  |
|  | Yes | 31 (38.8) | 9 (34.6) |  |
| Metastasis |  |  |  | 1.00 |
|  | No | 70 (87.5) | 23 (88.5) |  |
|  | Yes | 10 (12.5) | 3 (11.5) |  |
| Total lymph node extracted |  |  |  | 0.21 |
|  | Mean ± SD | 60.3 ± 27.4 | 68.9 ± 37.5 |  |
| No. of positive lymph node |  |  |  | 0.08 |
|  | Mean ± SD | 2.8 ± 3.8 | 1.7 ± 2.2 |  |
| Extra nodal extension |  |  |  | 0.06 |
|  | Absent | 71 (88.4) | 19 (73.1) |  |
|  | Present | 9 (11.3) | 7 (26.9) |  |
|  |  |  |  |  |

**Table S2: Demographic and clinicopathological characteristics versus PD-L1 (after chemotherapy) negative and positive.**

| Variables | Characteristics | Negative  75 (70.7%) | Positive  31 (29.2%) | p-value |
| --- | --- | --- | --- | --- |
| IDO after chemotherapy | |  |  | 0.007 |
|  | Negative | 62 (82.7) | 18 (58.1) |  |
|  | Positive | 13 (17.3) | 13 (41.9) |  |
| Age |  |  |  | 0.04 |
|  | Mean ± SD | 47.4 ± 11.4 | 52.5 ± 11.6 |  |
| Sex |  |  |  | 0.31 |
|  | Male | 48 (64.0) | 23 (74.2) |  |
|  | Female | 27 (36.0) | 8 (25.8) |  |
| Body mass index (kg/m^2^) |  |  |  | 0.76 |
|  | Mean ± SD | 26.0 ± 4.1 | 26.2 ± 5.3 |  |
|  |  |  |  |  |
| Family History |  |  |  | 0.34 |
|  | Absent | 67 (89.3) | 25 (80.6) |  |
|  | Present | 8 (10.7) | 6 (19.4) |  |
| History of Pan/Niswar |  |  |  | 0.86 |
|  | Absent | 52 (69.3) | 22 (71.0) |  |
|  | Present | 23 (30.7) | 9 (29.0) |  |
| History of tobacco use |  |  |  | 0.03 |
|  | Absent | 53 (73.3) | 16 (51.6) |  |
|  | Present | 20 (26.7) | 15 (48.4) |  |
| Comorbidities |  |  |  | 0.16 |
|  | Absent | 59 (78.7) | 26 (83.9) |  |
|  | Present | 16 (21.3) | 5 (16.1) |  |
|  | - DM | 4 (5.3) | - |  |
|  | - HTN | 7 (9.3) | 4 (12.9) |  |
|  | - DM + HTN | 5 (6.7) | - |  |
|  | - DM + Hepatitis C | - | 1 (3.2) |  |
| Differentiation |  |  |  | 0.21 |
|  | Poorly differentiated | 7 (9.3) | 3 (9.7) |  |
|  | Moderately differentiated | 45 (60.0) | 18 (51.1) |  |
|  | Well differentiated | 23 (30.7) | 10 (32.3) |  |
| Tumour site |  |  |  | 0.31 |
|  | Left | 42 (56.0) | 14 (45.2) |  |
|  | Right | 33 (44.0) | 17 (54.8) |  |
| Focality |  |  |  | 0.24 |
|  | Single | 74 (98.7) | 30 (96.8) |  |
|  | Bifocal | - | 1 (3.2) |  |
|  | Multifocal | 1 (1.3) | - |  |
| Margin status |  |  |  | 0.55 |
|  | Uninvolved | 72 (96.0) | 31 (100.0) |  |
|  | Involved | 3 (4.0) | - |  |
| Tumour size (mm) |  |  |  | 0.92 |
|  | Mean ± SD | 21.6 ± 11.6 | 21.3 ± 11.0 |  |
| DOI (mm) |  |  |  | 0.06 |
|  | Mean ± SD | 8.2 ± 4.4 | 10.1 ± 4.9 |  |
| T stage |  |  |  | 0.75 |
|  | 0 | 1 (1.3) | - |  |
|  | 1 | 20 (26.7) | 5 (16.1) |  |
|  | 2 | 30 (40.0) | 15 (48.4) |  |
|  | 3 | 22 (29.3) | 10 (32.3) |  |
|  | 4 | 2 (2.7) | 1 (3.2) |  |
| N stage |  |  |  | 0.55 |
|  | 0 | 35 (46.7) | 13 (41.9) |  |
|  | 1 | 12 (16.0) | 5 (16.1) |  |
|  | 2 | 18 (24.0) | 11 (35.5) |  |
|  | 3 | 10 (13.3) | 2 (6.5) |  |
| Recurrence |  |  |  | 0.89 |
|  | No | 47 (62.7) | 19 (61.3) |  |
|  | Yes | 28 (37.3) | 12 (38.7) |  |
| Metastasis |  |  |  | 0.33 |
|  | No | 64 (85.3) | 29 (93.5) |  |
|  | Yes | 11 (14.7) | 2 (6.5) |  |
| Total lymph node extracted |  |  |  | 0.36 |
|  | Mean ± SD | 60.7 ± 27.7 | 66.8 ± 37.0 |  |
| No. of positive lymph node |  |  |  | 0.71 |
|  | Mean ± SD | 1.8 ± 3.1 | 2.1 ± 3.3 |  |
| Extra nodal extension |  |  |  | 0.77 |
|  | Absent | 63 (84.0) | 27 (87.1) |  |
|  | Present | 12 (16.0) | 4 (12.9) |  |
|  |  |  |  |  |
